# General practitioner teachers’ job satisfaction and their medical students' wish to join the field – a correlational study

**DOI:** 10.1186/1471-2296-15-50

**Published:** 2014-03-24

**Authors:** Damian Notker Meli, Angie Ng, Sarah Singer, Peter Frey, Mireille Schaufelberger

**Affiliations:** 1 Berner Institut für Hausarztmedizin BIHAM, Gesellschaftsstrasse 49, 3012 Bern, Switzerland; 2 Durham University, 32 Old Elvet, Durham DH1 3HN, UK

**Keywords:** Clerkship, Teaching, General Practice, Job satisfaction, Medical students

## Abstract

**Background:**

There will be increasing competition for young physicians worldwide as more and more physicians retire. While enthusiasm towards GP work is important for GP teachers as role models, satisfaction within the profession has declined. This study aims to determine if medical students’ desire to become GPs is related to the job satisfaction of their teaching GPs and explore the factors tied to this job satisfaction.

**Methods:**

In this cross-sectional, correlational study, teaching GPs of the University of Bern and the fourth year medical students completing internships with them filled in separate questionnaires.

**Results:**

Whether or not the GP teacher is perceived by a student to be satisfied with her/his job is correlated to that student’s satisfaction with the internship, which in turn, is correlated with student’s wish to be a GP after the internship. Results show which factors are most related to GP job satisfaction and the effect of working hours and their composition.

**Conclusions:**

Medical students’ perception of their GP teachers’ job satisfaction positively affect their wish to become GPs, and their satisfaction with their internships adds to this. Enhancing the positive aspects of GP work, such as recognition, and improving negative ones, such as administrative duties, are necessary to attract medical students into the GP field.

## Background

Less and less students are interested in pursuing primary care careers, and the resulting shortage of General Practitioners (GPs) has also been noted in many countries around the world, including Germany [1,2], the United States [3,4] and Canada [5]. As more and more physicians retire, the competition for young physicians in Switzerland will increase [6].

Survey results in different countries indicate that satisfaction within the medical profession has declined in the past decades, a trend which also applies to other professions which have been increasingly constrained and corporatised by governmental and professional organisations, resulting in a loss of autonomy, status and public esteem [7]. Within the field of medicine, general practice has suffered from the primary care-specialty income gap [3] and relatively lower amount of associated prestige [4], and these problems need to be addressed in order to attract medical students.

It has been found that most medical students are open-minded towards working in General Practice [1], and it has been suggested that the job satisfaction of GPs affects the recruitment of new physicians into this field of medical practice [7]. In countries in which GP practices exist as independent businesses, the ability to work within an organisational setting rather than in their own practice positively influences students’ in the decision to become GPs [1]. Similarly, the existence of a professorship in General Practice [1], the improvement of family compatability and work-life balance for GPs [2,5]; and addressing concerns about its broad intellectual content’s being impossible to master [4,5] can also motivate students to become GPs. Studies suggest increasing the attractiveness of the field and marketing it to medical students early in medical school [2].

Research has demonstrated that positive role models influence the career choices of future physicians [8]. Clinical teachers are a key component in the ‘clinical learning environment’, and their using a learner-centred approach - being responsive to the needs of individual students, having more personal contact with them and giving them opportunities to participate in relevant activities - can positively influence student satisfaction [9]. As it has been suggested that the job satisfaction of GPs affects the recruitment of new physicians into this field of medical practice [7], it is imperative to determine if the job satisfaction of GPs who act as GP teachers is related to their medical students’ desire to join the field.

Research also suggests that exposing medical students to primary care settings encourages more students to choose careers in general practice, especially if they are given the opportunity to participate in health education [10]. GP teachers can influence their students by engaging them in their clinical work and by demonstrating enthusiasm towards their work [8].

There is a need for more research on the career choices medical school graduates make in Switzerland. Previous studies have found that it is necessary to promote the field to medical students early in their education [2], and a Swiss study of three German-speaking medical schools found that the majority of physicians have chosen their career paths before they are halfway through their residency [11]. So it is necessary to explore what factors can increase the number of medical students who will become GPs.

The context of this study is the medical school at the University of Berne. Medical education here consists of three years of Bachelor-level education followed by three years of Master-level education. In 2007, the medical school made it mandatory for all medical students to participate in a longitudinal, integrated clerkship in primary care [12]. Within this scheme, students are assigned a GP who acts as their mentor throughout the first four years of their studies; in the first three years of medical school, they spend eight half days per year with this GP, and in their fourth year, they stay with this GP for three weeks [12]. As this last period is the longest, it was chosen for this study.

The aims of this study are: (1) to determine whether or not medical students’ desire to become GPs is related to the job satisfaction of their GP teachers, and (2) to explore the factors tied to the job satisfaction of Swiss GP teachers.

## Methods

### Design, setting and sample

This cross-sectional, correlational study used questionnaires to obtain data from teaching GPs and their respective medical students. This study employed convenience sampling, recruiting only GP teachers associated with the University of Bern and the fourth-year medical students carrying out three-week internships with these GP teachers in 2012.

The students were requested to return questionnaires to the school upon completion by both themselves and their GP teachers. Participation was entirely optional.

### Variables and measurement

The survey instruments used self-assessment. These instruments can be found in the Additional file 1, and below is a brief explanation of the questionnaires utilised.

#### *Questionnaire for GPs*

The GP-questionnaire consists of 18 items, of which 11 were used in a summative scale to determine overall job satisfaction. This scale of 11 items used 10 items out of the original 15 of the Warr-Cook-Wall job satisfaction scale [13], which were chosen and modified by Cooper, Rout and Faragher especially for general practice [14], and the item added by Ulmer and Harris regarding government policy [15]. This scale has previously been validated in studies on physicians from Norway, Germany, the US, New Zealand and the UK [16]. Five additional self-developed items included working hours and other practice details. The remaining two items were the ones documenting gender and age.

#### *Questionnaires for students*

The questionnaire for medical students consisted of four items related to their internships and their desire to become GPs. These items were chosen as the most relevant to the research aims, and a shorter questionnaire was used to maximise the response rate.

### Data collection

The questionnaires completed by GPs were returned to their students in sealed envelopes, and these questionnaires were submitted along with the students’ at the end of the internships. The questionnaires were identified by a code, which allowed each student’s questionnaire to be matched with her/his teaching GP’s but not identification of individuals.

### Data analysis

The score resulting from the summative scale was used to determine GPs’ overall job satisfaction, while the corrected item-total correlation was used to determine the strength of each factor. Correlational analysis was used to detect any relationship between GP teacher’s job satisfaction and their student’s desire to become GPs. The data were analysed using SPSS v. 21.

### Ethics statement

The study is in compliance with the Helsinki Declaration. According to the cantonal ethical committee of Bern no full ethical approval was needed for this study.

## Results

There was a 53.8% response rate. 92 pairs of teacher-student questionnaires were returned out of the 171 pairs distributed.

### The influence on the medical students

The positive change in students’ desire to become GPs before and after their internship was highly significant (p < 0.0001) (Table 1). Whether or not the GP teacher is perceived by a student to be satisfied with her/his job is correlated to that student’s satisfaction with the internship (r_s_ = 0.520, significant to the 0.01 level), which in turn, is correlated with whether or not the student wishes to be a GP after the internship (r_s_ = 0.428, p < 0.01). The direct correlation between student perception of GP job satisfaction and and her/his own desire to enter the field after the internship was weaker (r_s_ = 0.255, p < 0.05).

**Table 1:** Student perception of GP satisfaction and impact on the internship

**Item**	**Mean score +/- standard deviation (Scale 1–7)**	**Correlation with total GP satisfaction score**	**Correlation with students perception of GP satisfaction**
Satisfaction with the clerkship	6.1 +/- 1.09	Ns	0.520, p < 0.01
Student’s perception of GP’s satisfaction with job	5.99 +/- 0.91	0.257, p < 0.05	1
Wanting to be a GP before clerkship	3.38 +/- 1.70	Ns	Ns
Wanting to be a GP after clerkship	4.26 +/- 1.47	Ns	0.255, p < 0.05

At the same time, the correlation between GP job satisfaction and student’s perception of this job satisfaction is lower (r_s_ = 0.257, p < 0.05). This suggests that there is a complex, indirect relationship between GP’s job satisfaction and students’ perception of their job satisfaction. Further studies are needed to understand more about this relationship.

**Figure 1:**
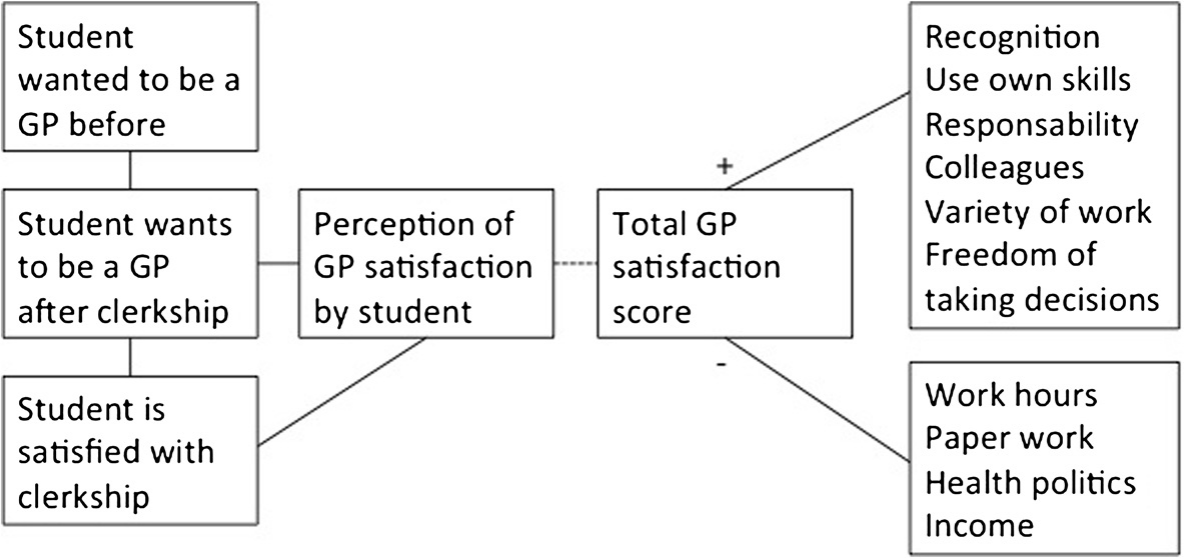
Correlations of GP job satisfaction and students’ wish to become a GP.

Figure 1 shows the correlation between various items.

The age range of the GP teacher was found to be negatively correlated with changes in whether or not the student wanted to become a GP before and after the internship (r_s_ = -0.296, p < 0.01).

### GP Job satisfaction and influencing factors

The summative scale has a Cronbach’s alpha of 0.836, confirming its reliability. Using the corrected item-scale correlation, we obtained a correlation ranking of the different items (Table 2), which shows the strength of correlation with GP job satisfaction. Although satisfaction with related government policies is correlated with the scale, it is only slightly so.

**Table 2:** Items and how they are correlated with the GP job satisfaction score

**Rank**	**Satisfaction with**	**Mean score +/- standard deviation (Scale 1–7)**	**Corrected correlation with total scale**
1	Satisfaction regarding all aspects	5.60 +/- 0.961	0.723
2	Recognition of your work	5.49 +/- 1.288	0.665
3	Opportunity to use own skills	6.17 +/- 0.897	0.627
4	Freedom of taking own decisions	6.16 +/- 0.986	0.625
5	Amount of responsibility given	5.70 +/- 1.070	0.604
6	Physical working conditions	4.93 +/- 1.305	0.504
7	Colleagues and practice staff	6.01 +/- 0.863	0.495
8	Working Hours	4.41 +/- 1.285	0.478
9	Variety	6.15 +/- 0.979	0.458
10	Income	5.08 +/- 1.303	0.448
11	Related government policies	2.95 +/- 1.287	0.192

The total GP satisfaction score was negatively correlated with actual hours per week (r_s_ = -0.376, p < 0.01). A GP’s satisfaction with her/his hours was negatively correlated with hours worked per week (r_s_ = -0.376, p < 0.01) and hours spent on paperwork per week (r_s_ = -0.244, p < 0.05) but not on-call hours per month.

The age range of GPs is negatively correlated with their satsifaction with colleagues and practice staff (r_s_ = -0.296, p < 0.01).

## Discussion

### The influence on the medical students

Results suggest that even a short experience in General Practice can have a positive effect on students’ wishes to become GPs, supporting previous studies [10]. The results show that there is a correlation between a medical student’s perception of a GP’s job satisfaction and whether or not the student wishes to become a GP; however, it appears that there is also a confounding variable in this relationship, that of the student’s satisfaction with the internship. This may indicate that, as prior studies suggest [8,10], GP teachers can act as positive role models not only by demonstrating job satisfaction but also in other ways, such as actively engaging students in clinical work.

Regarding the negative correlation between the GP teacher’s age and whether or not the student wants to become a GP after the internship, this could be explained by the existence of a generation gap between the student and the role model. This effect has been documented in previous research [5]. As GPs’ satisfaction with their colleagues and practice staff appears to decrease with age, perhaps it can also be hypothesised that the decrease in satisfaction with colleagues and practice staff also plays a role.

### GP Job satisfaction and influencing factors

The correlational analysis provides insights into the factors related to GP satisfaction, with GPs’ satisfaction with all aspects of their work being most important followed by their satisfaction for recognition received. Satisfaction with related government policies was least correlated; this is different from the results of other studies, such as ones carried out in the US [7], Australia [15] and New Zealand [17].

Hours worked per week, including that spent on paper work, and the satisfaction with these hours is significantly related to GPs overall job satisfaction. This supports previous studies carried out in other countries. For example, in the US, the UK and New Zealand, logistical and administrative tasks have been found to negatively impact professional satisfaction [7,14,17]. However, unlike the results from other countries, such as the UK [14] and New Zealand [17], this study’s results do not show on call work to be significantly, negatively correlated with job satisfaction. This study also did not uncover any significant findings related to gender despite previous findings showing importance of this factor [6].

Improving factors that increase GP job satisfaction is important in inspiring medical students to join the field, via both role modelling and marketing. As Switzerland’s need for new GPs is growing, it is necessary to enhance the positive aspects of GP work and improve other aspects to make it attractive to medical students. The same applies to other countries which are also experiencing a lack of medical students’ electing to become GPs.

### Limitations

Correlations and associations do not imply causation; they only show that a relationship exists between two items.

In addition, the small sample size taken from a single institution is also problematic, as is the low response rate.

Also, as the study did not ask the students to complete a questionnaire on their career preferences before their clerkships began, the results may reflect a recall bias. The questionnaire also did not inquire of students’ gender, which may have provided additional information.

### Recommendations for future research

More research needs to be done in Switzerland to see what factors in a role model, other than job satisfaction, can positively influence medical students to choose to become GPs. Further research is also necessary to examine the complex relationship between GP satisfaction and student perception of GP satisfaction, and whether or not GP teacher’s satisfaction with their teaching role is an important factor. Additional studies should also be carried out to determine what major factors influence medical students’ satisfaction with their internships, as this seems to be an important factor. Qualitative studies could also be carried out to further investigate the relationship between GP teachers’ age, their satisfaction with colleagues and practice staff and whether or not students wish to become GPs after their clerkships. Similar research should also be carried out in other countries which are interested in having more medical students become GPs in the future.

The high level of job satisfaction shown by GP teachers in this study also suggests that studies are needed to determine if GPs who elect to become GP teachers are actually more satisfied with their jobs than other GPs and what factors are involved. These may include gender, which has appeared in the literature [1] but did produce any significant findings in this particular research.

## Conclusion

This study has found a positive correlation between medical students' perception of their GP teacher's job satisfaction and the students' wish to become GPs. In order to attract more students into the GP field, it is necessary for government policies to enhance positive aspects of GP work, such as recognition, and improve negative ones, such as the amount of administrative duties. Furthermore, as medical students' satisfaction with their internships is also positively correlated with their desire to become GPs, it is important for GP teachers to act as positive role models.

## Competing interests

The authors declare that they have no competing interests.

## Authors’ contributions

DM: Involved in conception and design of the study, analysis and interpretation of the data, drafting the manuscript and given final approval to the version to be published. AN: Involved in design of the study, analysis of the data, drafting the manuscript, revising the manuscript and given final approval to the version to be published. SS: Involved in acquisition of data, revising the manuscript and given final approval to the version to be published. PF: Involved in acquisition of data, revising the manuscript and given final approval to the version to be published. MS: Involved in conception and design of the study, revising the manuscript and given final approval to the version to be published.

## Pre-publication history

The pre-publication history for this paper can be accessed here:

http://www.biomedcentral.com/1471-2296/15/50/prepub

## Supplementary Material

Additional file 1 Questionnaire for teaching GPs and questionnaire for medical students. Click here for file
