# Supplementary material for: General practitioner teachers’ job satisfaction and their medical students' wish to join the field – a correlational study
Source: BMC Fam Pract. 2014 Mar 24;15:50. doi: 10.1186/1471-2296-15-50 (PMC3997967; doi:10.1186/1471-2296-15-50)
Supplement: Additional file 1 — Questionnaire for teaching GPs and questionnaire for medical students. [file 1471-2296-15-50-S1.doc]

**Additional file 1**

Questionnaire for teaching GPs:

Please circle the answer to the following questions:

What gender do you identify with?: Female Male Other

What range does your age fall into?: 25-34 35-44 45-54

55-64 65 and above

How many doctors and assistant doctors work in your practice? 1 2 3+

What is your work setting? Rural Urban

Please fill in the answer in the blank provided after the question:

What is the approximate number of hours you work per week? _______

How many of your work yours, per week, are spent on paperwork? _______

How many hours per month do you do on-call work? _______

Please indicate how satisfied or dissatisfied you are with each of the various aspects of your job.

Please circle a response on the scale **1 - extremely dissatisfied**

**4-neutral**

**7 – extremely satisfied**

a) Your colleagues and fellow workers 1 2 3 4 5 6 7

b) Amount of responsibility you are given 1 2 3 4 5 6 7

c) Physical working conditions 1 2 3 4 5 6 7

d) Freedom to choose your own

method of working 1 2 3 4 5 6 7

e) Your income 1 2 3 4 5 6 7

f) Your hours of work 1 2 3 4 5 6 7

g) Opportunity to use your abilities 1 2 3 4 5 6 7

h) Recognition you get for good work 1 2 3 4 5 6 7

i) Related government policies 1 2 3 4 5 6 7

i) Amount of variety in your job 1 2 3 4 5 6 7

j) Taking everything into consideration,

what is your general feeling about the job? 1 2 3 4 5 6 7

Questionnaire for medical students:

Please indicate the extent to which you agree with the following statements after completing your 3-week internship in GP practice.

Please circle a response on the scale **1 – completely disagree 4 - neutral 7– completely agree**

a) I am very satisfied with my internship 1 2 3 4 5 6 7

b) I wanted to become a GP before my

internship 1 2 3 4 5 6 7

c) I want to become a GP now 1 2 3 4 5 6 7

d) My teaching GP is very satisfied with
 her/his job 1 2 3 4 5 6 7
